# A pilot study of genomic‐guided induction therapy followed by immunotherapy with difluoromethylornithine maintenance for high‐risk neuroblastoma

**DOI:** 10.1002/cnr2.1616

**Published:** 2022-03-31

**Authors:** Jacqueline M. Kraveka, Elizabeth C. Lewis, Genevieve Bergendahl, William Ferguson, Javier Oesterheld, Elizabeth Kim, Abhinav B. Nagulapally, Karl J. Dykema, Valerie I. Brown, William D. Roberts, Deanna Mitchell, Don Eslin, Derek Hanson, Michael S. Isakoff, Randal K. Wada, Virginia L. Harrod, Jawhar Rawwas, Gina Hanna, William P. D. Hendricks, Sara A. Byron, Matija Snuderl, Jonathan Serrano, Jeffrey M. Trent, Giselle L. Saulnier Sholler

**Affiliations:** ^1^ Medical University of South Carolina Charleston South Carolina USA; ^2^ Wayne State University School of Medicine Detroit Michigan USA; ^3^ Levine Children's Hospital, Atrium Health Charlotte North Carolina USA; ^4^ Saint Louis University School of Medicine St. Louis Missouri USA; ^5^ Wesleyan University Middletown Connecticut USA; ^6^ Penn State Children's Hospital at the Milton S. Hershey Medical Center and Penn State College of Medicine Hershey Pennsylvania USA; ^7^ Rady Children's Hospital San Diego and UC San Diego School of Medicine San Diego California USA; ^8^ Helen DeVos Children's Hospital at Spectrum Health Grand Rapids Michigan USA; ^9^ St. Joseph's Children's Hospital Tampa Florida USA; ^10^ Hackensack University Medical Center Hackensack New Jersey USA; ^11^ Center for Cancer and Blood Disorders Connecticut Children's Medical Center Hartford Connecticut USA; ^12^ Kapiolani Medical Center for Women & Children Honolulu Hawaii USA; ^13^ Dell Children's Medical Center Austin Texas USA; ^14^ Children's Hospitals and Clinics of Minnesota Minneapolis Minnesota USA; ^15^ Orlando Health Cancer Institute Orlando Florida USA; ^16^ Translational Genomics Research Institute (TGen) Phoenix Arizona USA; ^17^ NYU Langone Health and NYU Grossman School of Medicine New York City New York USA

**Keywords:** DFMO, immunotherapy, maintenance, neuroblastoma, precision medicine

## Abstract

**Background:**

Survival for patients with high‐risk neuroblastoma (HRNB) remains poor despite aggressive multimodal therapies.

**Aims:**

To study the feasibility and safety of incorporating a genomic‐based targeted agent to induction therapy for HRNB as well as the feasibility and safety of adding difluoromethylornithine (DFMO) to anti‐GD2 immunotherapy.

**Methods:**

Twenty newly diagnosed HRNB patients were treated on this multicenter pilot trial. Molecular tumor boards selected one of six targeted agents based on tumor‐normal whole exome sequencing and tumor RNA‐sequencing results. Treatment followed standard upfront HRNB chemotherapy with the addition of the selected targeted agent to cycles 3–6 of induction. Following consolidation, DFMO (750 mg/m^2^ twice daily) was added to maintenance with dinutuximab and isotretinoin, followed by continuation of DFMO alone for 2 years. DNA methylation analysis was performed retrospectively and compared to RNA expression.

**Results:**

Of the 20 subjects enrolled, 19 started targeted therapy during cycle 3 and 1 started during cycle 5. Eighty‐five percent of subjects met feasibility criteria (receiving 75% of targeted agent doses). Addition of targeted agents did not result in toxicities requiring dose reduction of chemotherapy or permanent discontinuation of targeted agent. Following standard consolidation, 15 subjects continued onto immunotherapy with DFMO. This combination was well‐tolerated and resulted in no unexpected adverse events related to DFMO.

**Conclusion:**

This study demonstrates the safety and feasibility of adding targeted agents to standard induction therapy and adding DFMO to immunotherapy for HRNB. This treatment regimen has been expanded to a Phase II trial to evaluate efficacy.

## 1 INTRODUCTION

Neuroblastoma (NB) accounts for 7%–10% of childhood cancer diagnoses but 15% of all pediatric cancer deaths in the United States.^1^, ^2^ Patients diagnosed with high‐risk NB (HRNB) have survival rates of ~40%–50%,^3^, ^4^ despite intensification of upfront therapy including^5^, ^6^, ^7^ 5–6 cycles of induction chemotherapy, surgical resection, consolidative myeloablative chemotherapy followed by autologous peripheral blood stem cell transplantation (auto‐PBSCT), radiation therapy (XRT), and maintenance therapy utilizing anti‐GD2 immunotherapy with dinutuximab combined with isotretinoin. A retrospective study of HRNB patients showed that those who did not achieve at least a partial response at the end of induction demonstrated significantly poorer event‐free survival (EFS) and overall survival (OS).^8^ Currently, the standard treatment within the Children's Oncology Group (COG) includes 5 cycles of induction chemotherapy followed by tandem high‐dose chemotherapy and stem cell transplantation. In addition, the current COG study (ANBL1531) performs targeted genomic sequencing for the incorporation of crizotinib, an *ALK* inhibitor, for HRNB patients with *ALK* aberrations. Following induction, the COG has shown superiority of tandem transplant over a single Carboplatin/Etoposide/Melphalan (CEM) transplant,^6^ while the Society of Pediatric Oncology European Neuroblastoma Network (SIOPEN) has shown superiority of Busulfan‐Melphalan transplant over single CEM transplant following their induction regimen.^9^ Further research and improvements in upfront therapy are needed.

Since the demonstration of successful targeted anti‐cancer therapy over 20 years ago,^10^ many targeted therapies that exploit molecular aberrations in cancer cells have been developed. While the incorporation of biologically relevant targeted agents to chemotherapy has the potential to significantly improve induction response rates and overall outcomes, applicability to clinical care has been modest at best. However, some recent successes include the activity of larotrectinib^11^ in *NTRK* translocated tumors and crizotinib^12^ in the setting of activating *ALK* mutations, independent of tissue origin or histology. We hypothesized that incorporation of novel targeted agents into standard induction therapy would improve induction responses in HRNB.Even in HRNB patients who respond well to initial therapy, the risk of relapse remains substantial, and long‐term survival following relapse is dismal. One strategy to prevent relapse is use of maintenance therapy, such as in a recent study in rhabdomyosarcoma.^13^ The ornithine decarboxylase (ODC) inhibitor difluoromethylornithine (DFMO) has been shown to reduce the highly tumorigenic CD114+ subpopulation as well as neurosphere formation and tumor initiation in vitro and in vivo.^14^ As previously reported, DFMO given as maintenance therapy for 2 years following completion of standard therapy was associated with significantly improved EFS and OS at 2‐ and 5‐years.^15^, ^16^ We hypothesized that incorporation of DFMO at the beginning of immunotherapy as an early relapse prevention strategy might lead to a more durable remission with improved EFS and OS in patients with HRNB.

Herein, we report on the feasibility and safety of adding a molecularly‐based targeted agent to induction chemotherapy and DFMO to immunotherapy for HRNB patients.

## 2 METHODS

### 2.1 Objectives

The primary objectives of this study were to determine the feasibility of incorporating a targeted agent identified by molecular profiling into cycles 3–6 of standard HRNB induction therapy, and to assess the feasibility and safety of adding DFMO to immunotherapy and subsequent continuation of DFMO for 2 years (or until tumor progression).

Secondary objectives included acute toxicity monitoring, response to treatment as measured by overall response rate (ORR) after induction, and EFS. Tumor epigenetics were studied as an exploratory objective.

### 2.2 Study design & patient selection

This pilot study was a prospective, open label, multicenter clinical trial. It was approved by the Western Institutional Review Board (WIRB) as well as by all local institutional review boards (IRB) at participating Beat Childhood Cancer (BCC) sites. Consent for study participation was obtained from all subjects according to federal and institutional guidelines. ClinicalTrials.gov Identifiers: NCT02559778.

Inclusion criteria included: diagnosis of HRNB as defined by Children's Oncology Group criteria^17^ or ganglioneuroblastoma (nodular or intermixed) by histology or presence of NB in bone marrow with elevated urine catecholamines; age ≤ 21 years at initial diagnosis; no prior systemic therapy (except for localized emergency radiation to sites of life‐ or function‐threatening disease and/or no more than 1 cycle of chemotherapy per a low‐ or intermediate‐risk neuroblastoma regimen); and adequate liver, renal, and cardiac function.

### 2.3 Sample processing and analysis

Tumor‐normal whole exome sequencing (WES) and tumor RNA‐sequencing (RNA‐Seq) were performed as previously described^18^ at Ashion Analytics (http://www.ashion.com), a CAP‐accredited, CLIA‐certified laboratory. Data were aligned to build 37 of the human reference genome. The mean target exome coverage was 461X for tumor samples and the average number of tumor RNA mapped reads was 98 M.

Each subjects' genomic data were reviewed at a molecular tumor board, wherein a specific targeted agent was selected from a panel of agents (bortezomib, crizotinib, dasatinib, lapatinib, sorafenib, and vorinostat) derived from prior WES and RNA‐Seq analyses of 48 pediatric NB subjects. Each of these agents had been tested in pediatric phase I and II studies and had established pediatric dosing.^12^, ^19^, ^20^, ^21^, ^22^ Actionable DNA alterations, defined as alterations with literature evidence supporting an association with response to the targeted agents in this study, were given priority in choosing the targeted agent. However, due to the low mutational burden, RNA expression was heavily relied upon for drug selection.

### 2.4 Somatic variant analysis

Seurat was used for calling somatic single nucleotide variants and small indels. A custom read depth‐based comparative method (https://github.com/tgen/tCoNuT) was used for detecting copy number variants. Manta was used for structural variant calling. TopHat fusion was used for fusion detection, as previously described.^23^

### 2.5 RNA expression analysis

Tumor RNA expression levels were compared to a normal whole body reference panel composed of 22 commercially purchased samples representing 14 normal tissues (adrenal gland, brain, bronchus, esophagus, heart, large intestine, liver, lung, lymph node, pituitary gland, skeletal muscle, skin, spleen, and uterus). The results were represented as a *Z*‐score, a measure of relative expression of genes in tumor versus normal reference, as described previously.^24^ Tumor RNA expression levels were also compared to expression in a panel of 41 HRNB tumors, and results represented as a cumulative percentage relative to other HRNB tumors. *Z* scores > 2 (RNA expression value in a patient's tumor was two standard deviations above the mean value for normal tissues) and cumulative cancer reference scores >0.75 (RNA expression value in a patient's tumor was in the top quartile compared to other HRNB tumors) that matched to preset expression‐based drug rules for this study were selected for discussion within the molecular tumor board. Data were submitted to a database of algorithms designed to predict relevant medications which were then presented in a report to the molecular tumor board. These algorithms included: biomarker rules, drug target expression, network‐based methods, drug response, and drug sensitivity signatures.^18^

### 2.6 Methylation

DNA methylation profiling was performed on the tumor samples, retrospectively, by treating the DNA with sodium metabisulfite and scanning the treated DNA on Illumina Human Methylation Infinium EPIC microarrays as described previously.^25^, ^26^, ^27^ Only probes in the promoter region of each gene were used to calculate the average beta value per gene, as the methylation status in the promoter region of each gene has the strongest correlation to gene expression.^28^

### 2.7 Treatment

**FIGURE 1:**
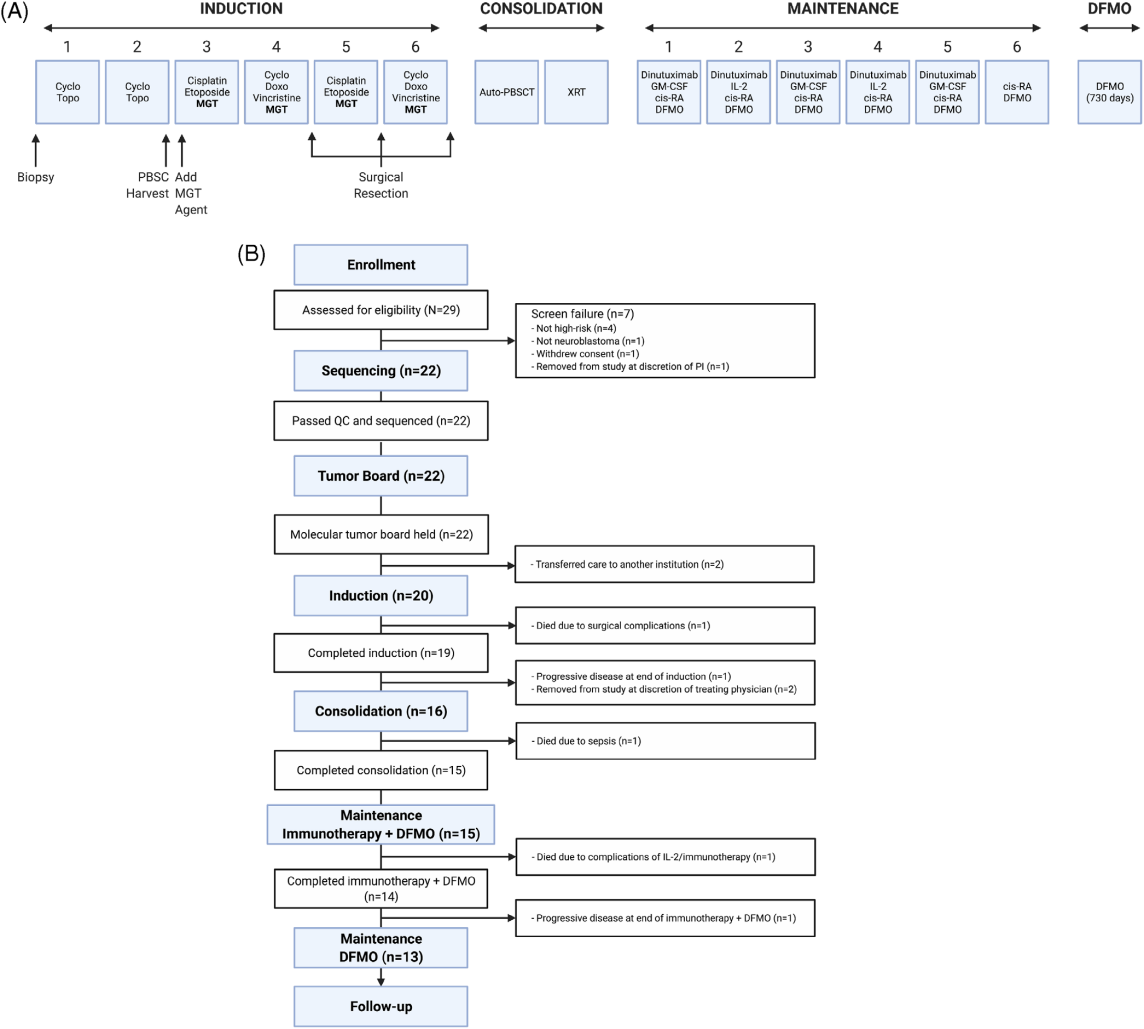
(A) NMTRC012 study flow diagram. (B) Consort diagram of NMTRC012 pilot

**FIGURE 2:**
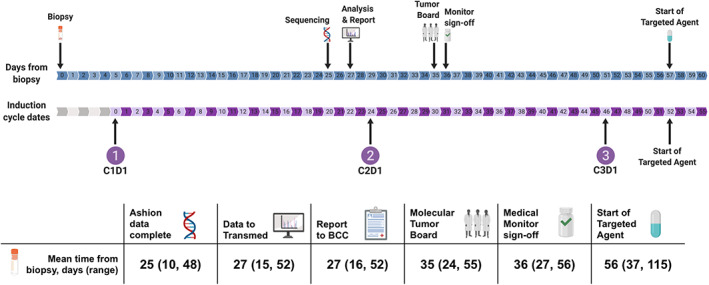
Feasibility timeline shows mean days from biopsy to study timepoints including completion of genomic sequencing, receipt of analysis and report by beat childhood cancer (BCC) team, convening of molecular tumor board, medical monitor sign‐off, and initiation of targeted therapy. Feasibility timeline is overlayed by mean induction cycle start days. Corresponding table provides mean days with ranges from biopsy to study timepoints

Induction therapy included six cycles of chemotherapy following a standard upfront therapy backbone.^6^, ^7^ Cycles were 21 days in duration (Figure 2B). The selected targeted agent was added to cycles 3–6, following collection of peripheral blood hematopoietic stem cells. Surgical resection of the primary tumor (if needed) could occur after cycles 4, 5, or 6. Consolidation therapy consisted of high‐dose busulfan/melphalan with auto‐PBSCT, followed by radiation therapy. Maintenance therapy included five cycles of dinutuximab plus granulocyte‐macrophage colony‐stimulating factor (GM‐CSF) alternating with interleukin‐2 (IL‐2) and six cycles of isotretinoin, as per ANBL0032.^29^ Oral DFMO, 750 mg/m^2^ twice daily, was started at the initiation of maintenance and continued until 2 years following the completion of cycle 6 of isotretinoin (Figure 1A).^30^

Toxicity analysis was conducted on all subjects who received at least one dose of targeted therapy and included all reported expected and unexpected adverse events, laboratory abnormalities, and the frequency of dose interruptions, dose reductions and treatment discontinuation. Toxicity was assessed at each clinical visit which included a physical exam as well as a CBC and CMP. Audiology was assessed at the start of DFMO, end of Cycle 3 and 6 of immunotherapy, and at Days 181, 361, and 730 of DFMO alone. Adverse events were graded according to the Common Terminology Criteria for Adverse Events (CTCAE) v5.0.

Subjects were required to have an ANC of ≥750/μl and platelets ≥75 000/μl prior to starting each induction cycle. The dose of targeted therapy was reduced by 25% if hematopoietic recovery delay was attributable to the agent. Delays were deemed attributable when >7 days. The doses of chemotherapy (except vincristine) were reduced by 25% if count recovery occurred between days 30–43 and by 50% if count recovery occurred after day 43. During immunotherapy/maintenance, dose reductions of DFMO were permitted for adverse events ≥grade 3.

Response was measured using the 1993 International Neuroblastoma Risk Group (INRG) criteria^3^ at standard time points including end of induction, end of consolidation, after 3 cycles of immunotherapy, at end of immunotherapy, every 3 months during the first 6 months of DFMO monotherapy, then every 6 months until completion of DFMO monotherapy.

## 3 RESULTS

### 3.1 Patient characteristics

Twenty subjects were evaluated for feasibility and safety. Table 1 and Table S1 detail subject characteristics. The median age at diagnosis/enrollment was 3 years (range: 0.25–14 years). Sixty percent of subjects were male and 65% self‐identified as White; 80% had stage 4 disease, with the remaining 20% having stage 3 disease. *MYCN* amplification was present in 45% of tumors, 90% had unfavorable histology, and 50% had a DNA index of 1 (diploid).

**TABLE 1:** Patient characteristics

NMTRC012 pilot	*N* = 20
Patient characteristics	
Age, years	
Mean	3.65
Median	3
Range	0.25–14
Sex, *n* (%)	
Male	12 (60%)
Female	8 (40%)
Race, *n* (%)	
Black/African American	5 (25%)
Multiracial	2 (10%)
White	13 (65%)
Stage at diagnosis, *n* (%)	
Stage 3	4 (20%)
Stage 4	16 (80%)
MYCN, *n* (%)	
Amplified	9 (45%)
Non‐amplified	11 (55%)
Histology, *n* (%)	
Unfavorable	18 (90%)
Favorable	2 (10%)
DNA index, *n* (%)	
>1 (Hyperdiploid)	4 (20%)
=1 (Diploid)	10 (50%)
Unknown	6 (30%)

### 3.2 Feasibility

Figure 1A,B depict the study flow diagram and Consort diagram, respectively. Twenty‐nine subjects were enrolled on the pilot study and assessed for eligibility. Seven of the 29 failed screening. The remaining 22 underwent diagnostic biopsies and analysis by WES and RNA‐Seq. Of the biopsies performed, 20 were from primary tumor site (12 abdominal, seven retroperitoneal, one paraspinal) and two were from metastases (one bone marrow, one supraclavicular lymph node). All 22 tumor samples passed QC thresholds and underwent sequencing. The mean time from biopsy to receipt of final report of DNA exome and RNA‐seq data was 27 days (range: 16–52 days) (Figure 2). Molecular tumor boards were conducted prior to the planned start of cycle 3 for all 22 subjects who underwent sequencing, with a mean time from biopsy to molecular tumor board review and target therapy recommendation of 35 days (range: 24–55 days) (Figure 2). Overexpression of HDAC*s* (*HDAC2* and/or *HDAC9*) and *ALK* were seen in the majority of tumors. The tumor board selected an HDAC inhibitor in a majority of cases (Table 2, Table S4).

**TABLE 2:** Molecular tumor board drug selection

Patient	Drug	Gene mutation	Gene overexpression
SL00402	VORINOSTAT		HDAC2, HDAC4, HDAC9
SL00393	VORINOSTAT		HDAC 2, HDAC8, RAD 23, CTBP2
SL00477	VORINOSTAT		HDAC 2, HDAC9, CTBP2
SL00522	CRIZOTINIB	ALK CVN gain	ALK
SL00511	SORAFENIB		RET
SL00535	DASATINIB		PDGFRB, DDR2
SL00545	VORINOSTAT		HDAC2
SL00558	VORINOSTAT		HDAC2
SL00575	VORINOSTAT		HDAC2
SL00581	VORINOSTAT		HDAC2, HDAC9
SL00586	VORINOSTAT		HDAC2, HDAC9
SL00587	VORINOSTAT		HDAC2, HDAC9
SL00589	VORINOSTAT		HDAC2, HDAC9
SL00605	CRIZOTINIB	ALK SNV F1174L	ALK
SL00608	VORINOSTAT		HDAC2, HDAC8, CTBP2
SL00625	VORINOSTAT		HDAC2, HDAC9
SL00650	VORINOSTAT		HDAC2
SL00653	VORINOSTAT		HDAC2
SL00680	VORINOSTAT		HDAC2
SL00720	VORINOSTAT		HDAC2

Two subjects chose to transfer to other institutions and did not proceed on study. The remaining 20 subjects continued on study and received induction chemotherapy. The most common targeted agent chosen was vorinostat (16 of 20), followed by crizotinib (2), dasatinib (1), and sorafenib (1) (Table 3). Nineteen subjects started targeted therapy during cycle 3, with 15 of those able to start on cycle 3 day 1. Initiation of targeted therapy was markedly delayed for only one patient, who started during induction cycle 5, due to a delay in obtaining insurance coverage. Eighty‐five percent of subjects met the feasibility definition of receiving 75% of targeted agent doses. Three subjects received <75% of doses: one due to toxicity (holds during febrile neutropenia), one due to parental refusal to give targeted agent, and one due to the aforementioned insurance approval delay.

**TABLE 3:** Targeted agent feasibility & safety profile

NMTRC012 pilot	*N* = 20
Targeted agent feasibility & safety profile	
Tumor board recommended agent, # subjects (%)	
Vorinostat (230 mg/m^2^/dose oral daily)	16 (80%)
Crizotinib (165 mg/m^2^/dose oral twice daily)	2 (10%)
Dasatinib (60 mg/m^2^/dose oral daily)	1 (5%)
Sorafenib (150 mg/m^2^/dose oral twice daily)	1 (5%)
Start of targeted agent, # subjects (%)	
Cycle 3, Day 1	15 (75%)
Cycle 3, after Day 1	4 (20%)
Cycle 5, Day 1	1 (5%)
Toxicity‐associated events, # subjects (%)	
Targeted agent holds	8 (40%)
Targeted agent dose reductions	2 (10%)
Targeted agent discontinuation	0 (0%)
Cycle delays (>7 days)	7 (35%)
Completed cycles of induction, # subjects (%)	
Six cycles	19 (95%)
Five cycles	1 (5%)
Feasibility of targeted agent	
Completed ≥75%	17 (85%)
Completed <75%	3 (15%)

The feasibility of adding DFMO to dinutuximab followed by 2 years of DFMO alone was also assessed. Fifteen patients were able to begin therapy with DFMO in combination with immunotherapy (70%), and 14 of the 15 were able to complete immunotherapy. Thirteen of these proceeded to DFMO monotherapy.

### 3.3 Safety

Grade 3–5 adverse events reported during cycles 3–6 of induction and at least possibly related to the targeted agent are summarized in Table 4. Expected adverse events solely related to standard chemotherapy, and not at least possibly related to the addition of targeted agent, were not reported. The most frequent adverse event was thrombocytopenia (70%) followed by anemia and neutropenia. The most common non‐hematologic adverse events were electrolyte abnormalities. Of the 20 subjects, eight experienced a temporary targeted agent hold, two experienced a targeted agent dose reduction of 25%, and seven had a delay of cycle initiation >7 days. The patients who did not have a dose reduction were not delayed secondary to targeted agent therefore a dose reduction was not warranted. Other reasons for cycle delays included: recovery from surgical resection, recovery from mucositis, recovery from febrile neutropenia, or recovery from count drop unrelated to targeted agents. None of the subjects required dose reduction of standard chemotherapy or permanent discontinuation of the targeted agent. There were no adverse events greater than or equal to grade 3 attributed to DFMO. For patients who remained on study to begin DFMO, seven had pre‐DFMO Grade 3 hearing toxicity, four had Grade 2, and three had normal hearing. No patients experienced worsening of hearing loss related to DFMO.

**TABLE 4:** Adverse events during induction therapy with targeted agent

NMTRC012 pilot	*N* = 20		
Adverse events^a^, cycles 3–6	Grade 3	Grade 4	Grade 5
Hematologic toxic effects, *n* (%)			
Anemia	9 (45%)	1 (5%)	
Lymphocytopenia		1 (5%)	
Neutropenia	2 (10%)	8 (40%)	
Febrile neutropenia	7 (35%)	4 (20%)	
Thrombocytopenia	2 (10%)	12 (60%)	
Leukopenia	2 (10%)	5 (25%)	
Non‐hematologic toxic effects, *n* (%)			
Elevated ALT	1 (5%)		
Elevated AST	1 (5%)		
Anorexia	1 (5%)		
Cellulitis	1 (5%)		
Dehydration	1 (5%)		
Diarrhea	1 (5%)		
Epistaxis	2 (10%)		
Hypophosphatemia	5 (25%)		
Hypokalemia	5 (25%)	1 (5%)	
Hyponatremia	3 (15%)		
Infection	3 (15%)	1 (5%)	
Nausea	4 (20%)		
Sepsis	1 (5%)	2 (10%)	
Skin infection		1 (5%)	
Thromboembolic event	1 (5%)		
Vomiting	3 (15%)		
Weight loss	1 (5%)		

a Expected and unexpected adverse events related to targeted agent.

Three subjects died during this trial: one due to complications during surgical resection, one from sepsis during consolidation, and one from brain herniation due to complications attributed to IL‐2 and dinutuximab on day 44 of immunotherapy.

### 3.4 Response

Of the 19 subjects who completed induction therapy, there were four complete remissions (CR) (21.1%), seven very good partial remissions (VGPR) (36.8%), six partial remissions (PR) (31.6%), and one each of mixed response (MR) (5.3%) and progressive disease (PD) (5.3%). The subject who progressed following induction therapy was ineligible to continue on study. The 18 subjects who showed at least a mixed response to induction were permitted to continue onto consolidation, although two subjects were removed from study at the end of induction per treating physician discretion, and one subject died during consolidation. Fifteen subjects subsequently continued to immunotherapy with dinutuximab, isotretinoin, and DFMO. Fourteen subjects completed immunotherapy and were evaluable for response, as one died during immunotherapy. Of those evaluable, 5 (35.7%) achieved CR, 5 (35.7%) VGPR, and 3 (21.4%) showed PR at the completion of immunotherapy. One subject progressed following immunotherapy and was ineligible to receive the 2 years of DFMO alone. The remaining 13 subjects went on to receive 2 years of maintenance therapy with DFMO. Final responses will be available once all subjects have completed DFMO monotherapy.

### 3.5 Genomics

**FIGURE 3:**
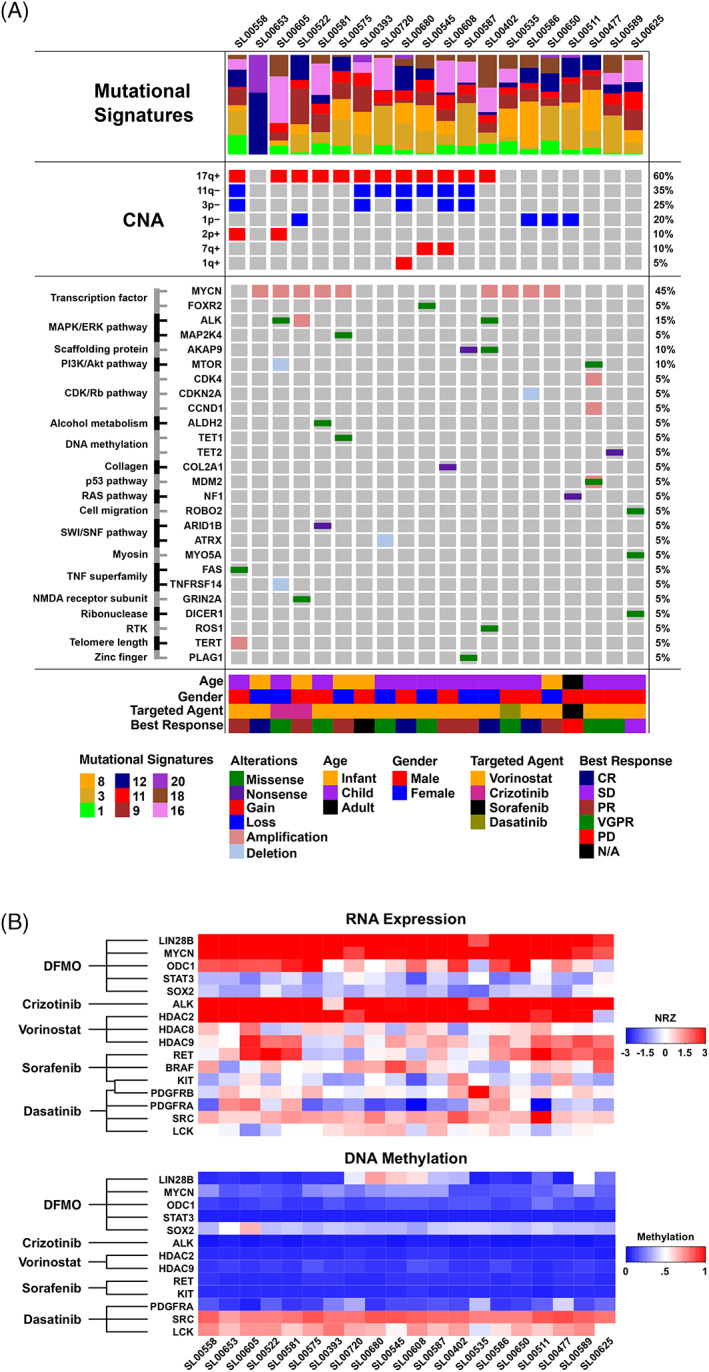
(A) Oncoprint showing genomic, phenotypic, and clinical notes of interest. Genomic features from top to bottom include mutational signatures (Sanger COSMIC v2), recurrent large‐scale copy number alterations (CNA), and small‐scale genomic alterations including mutations with focal copy number alterations. Age, gender, targeted agent chosen at tumor board, and best response seen in the patient are also shown. (B) Heatmap showing RNA expression and DNA methylation of key drug target genes. Drug names are shown on the left with lines indicating which genes are targeted by each therapeutic. The top panel shows expression as an NRZ score (normal *Z*‐score) relative to a panel of 22 normal tissues. Darker red indicates overexpression (NRZ > 3), white indicates normal expression status (NRZ = 0), and darker blue indicates underexpression (NRZ < −3). The bottom panel shows methylation scores as beta values. Darker red indicates hypermethylation (beta>0.80), white indicates neutral methylation status at the loci (beta ~ 0.5), and darker blue indicates hypomethylation at the gene loci (beta < 0.20). *BRAF* and *PDGFRB* were omitted from the methylation heatmap as these genes did not contain methylation probes within the TSS200 region that passed the probe quality criteria. *HƒDAC8* was also omitted as this gene had methylation probes within the promoter region, but farther upstream and not annotated within TSS200

WES and RNA‐Seq analyses were performed on all 20 tumor samples included for evaluation in this study. The overall mutational burden was low. Mutations and copy number alterations most frequently seen in cancer are plotted in the oncoprint (Figure 3A, Table S2). As expected, *MYCN* amplification (45%) and *ALK* alterations (15%) were the most frequently observed genetic aberrations. Several segmental changes were also identified, including large‐scale gains in chromosomes 1q, 2p, 7q, and 17q and losses in 1p, 3p, and 11q.^31^ RNA‐Seq results were also considered during molecular tumor board deliberations as one line of evidence for choosing a targeted therapy. *MYCN*, *LIN28B*, *ODC1*, and *HDAC2* were overexpressed in the majority of tumors. Of interest, the gene target for DFMO, *ODC1*, was only overexpressed in about half of the tumors but the downstream target, *LIN28B*, was overexpressed in all tumors.

COSMIC Mutational signatures (https://cancer.sanger.ac.uk/cosmic/signatures_v2) (version 2) were created for each tumor sample and are shown in the oncoprint (Figure 3A, Table S3), along with RNA profiles of targets and methylation patterns potentially relevant to trial therapeutics (Figure 3B, Table S4). COSMIC mutational signature 18 is frequently reported in neuroblastoma and was seen in 15 of 20 samples. It has been previously shown to be related to damage by reactive oxygen species,^32^, ^33^ which is a known biological stimulus in neuroblastoma.^34^

### 3.6 Methylation

Figure 3B shows a heatmap of methylation analysis performed on the tumors. Methylation results correlated with RNA expression, with the majority of overexpressed genes also showing hypomethylation. Of the genes relating to the targeted agents used in this study, *SRC* and *LCK* were the only ones noted to be hypermethylated. *SOX2* displayed slight hypomethylation across samples. The remaining genes, *PDGFRA*, *KIT*, *RET*, *HDAC9*, *ALK*, *HDAC2*, and *STAT3* were consistently hypomethylated. LIN28B was associated with variable methylation among samples as the majority of the cases in the heatmap were hypomethylated, with the exception of three samples appearing as slightly hypermethylated. (Figure 3B, Table S5).

## 4 DISCUSSION

This multicenter, prospective pilot study represents a strategy for the management of newly diagnosed HRNB, adding individualized therapy identified by molecular profiling to standard induction chemotherapy as well as the addition of DFMO to immunotherapy and extended maintenance monotherapy.

It proved feasible to perform molecular profiling of tumor samples, followed by a multi‐institutional, multidisciplinary tumor board to choose a specific targeted agent to be incorporated by cycle 3 of induction. All 22 eligible subjects were able to have tissue processed, although two subjects elected to transfer care to other institutions and did not proceed with protocol therapy. Of the twenty subjects who received the assigned targeted agent, 95% were able to start the agent during cycle 3. Although significant variation in the length of time needed to obtain insurance approval for targeted agents was noted, only one patient experienced a prolonged delay in starting due to delayed insurance authorization. Of note, 80% of patients were selected to receive vorinostat as the targeted agent. This medication has been studied in neuroblastoma in preclinical studies^35^, ^36^ as well as in clinical trials^37^ showing the histone deacetylase pathway to be important in neuroblastoma and targeting this may be beneficial. Of note, a recent study combining vorinostat with MIBG therapy for neuroblastoma has shown the addition of vorinostat to improve response rates relative to MIBG alone.^38^

The genomics of this patient cohort closely matched previously published neuroblastoma studies. *MYCN* amplification (45%) and *ALK* alterations (15%) were the most frequently observed findings. Of note, while <50% of the tumors showed DNA alterations in these genes, >90% of the subjects showed overexpression suggesting these genes may be playing a role in oncogenesis of HRNB, even in the absence of detected DNA alterations. Several segmental changes indicative of chromosomal instability were also identified, consistent with previous reports.^31^

There were no unexpected adverse events related to the addition of a targeted agent to standard induction therapy. All adverse events reported during induction were expected based on the established safety profile of the agents,^6^ and our overall observed rates of adverse events were comparable to a similar pilot study that evaluated the addition of topotecan and cyclophosphamide to induction therapy.^7^ Thrombocytopenia was the most frequent toxicity, observed at a frequency similar to other published reports of induction therapy in HRNB.^6^ Non‐hematologic toxicities were those expected with this chemotherapy backbone and were all manageable. Therefore, the addition of a targeted agent to standard induction therapy did not lead to substantial delays or dose reductions of conventional chemotherapy or an increase in toxic deaths.

It is notable that three of the 20 subjects died due to toxicities attributable to standard of care therapy. Although none of the patient deaths can be directly attributable to the targeted drugs added to induction treatment, there is the potential for late toxicity from these novel drug combinations. All subjects who died on study received vorinostat as their targeted agent, however, as noted above, 80% of the subjects on this trial received vorinostat. The size of this pilot study is not sufficient to make any statistical inference at this time, but additional data will be collected on the expansion study.

This pilot study was limited by the small number of enrolled patients and a lack of randomization; thus, meaningful statistical assessments of response and toxicity data were not possible. However, the observation that response rates were at least equivalent to those previously reported in similar patient populations justifies further exploration of the efficacy of adding targeted agents to induction chemotherapy. In addition, there were no reported adverse events (grade ≥ 2) related to the administration of DFMO during and after immunotherapy, consistent with prior reports that DFMO is well tolerated as maintenance therapy following immunotherapy.^15^, ^16^ While a potential toxicity of DFMO is hearing impairment, which was observed in a Phase II study with DFMO in neuroblastoma patients at a rate of <5%,^16^ this toxicity was not observed in patients on this trial. Of note, the dose used in this trial was 1500 mg/m^2^/day while other trials have used doses as high as 6750 mg/m^2^/day.^39^ It is important to note that hearing loss is a dose dependent toxicity of DFMO, hence the paucity of this toxicity on this study.

## 5 CONCLUSION

While advances in our understanding of the molecular pathways controlling tumor initiation, proliferation, and survival have led to the development of multiple new drugs with potential anti‐tumor effect, optimal incorporation of these agents as substitutes for, or adjuncts to, standard therapy remains largely undefined. This study evaluated the feasibility and safety of incorporating a targeted agent selected by WES and gene expression analysis into standard induction therapy for HRNB, as well as the addition of the ODC inhibitor DFMO during and after immunotherapy. While limited by the small number of subjects, both interventions were feasible and did not appear to add toxicity to standard therapy. An expansion study is underway to further evaluate the efficacy and safety of adding targeted agents to induction therapy, as well as the randomized addition of DFMO either at the beginning or end of immunotherapy.

## CONFLICT OF INTEREST

The authors declare no potential conflicts of interests.

## AUTHOR CONTRIBUTIONS

**Jacqueline M. Kraveka:** Conceptualization (equal); formal analysis (equal); investigation (equal); methodology (equal); writing – original draft (equal); writing – review and editing (equal). **Elizabeth C. Lewis:** Data curation (equal); formal analysis (equal); writing – original draft (equal); writing – review and editing (equal). **Genevieve Bergendahl:** Conceptualization (equal); data curation (equal); formal analysis (equal); methodology (equal); project administration (equal); writing – original draft (equal); writing – review and editing (equal). **William Ferguson:** Conceptualization (equal); formal analysis (equal); investigation (equal); methodology (equal); writing – original draft (equal); writing – review and editing (equal). **Javier Oesterheld:** Conceptualization (equal); formal analysis (equal); investigation (equal); methodology (equal); writing – original draft (equal); writing – review and editing (equal). **Elizabeth Kim:** Investigation (equal); writing – original draft (equal). **Abhinav B. Nagulapally:** Conceptualization (equal); formal analysis (equal); methodology (equal); software (equal); validation (equal); writing – original draft (equal); writing – review and editing (equal). **Karl J. Dykema:** Conceptualization (equal); formal analysis (equal); methodology (equal); software (equal); validation (equal); writing – original draft (equal); writing – review and editing (equal). **Valerie I. Brown:** Conceptualization (equal); formal analysis (equal); investigation (equal); methodology (equal); writing – original draft (equal). **William D. Roberts:** Conceptualization (equal); investigation (equal); methodology (equal); writing – original draft (equal). **Deanna Mitchell:** Conceptualization (equal); investigation (equal); methodology (equal); writing – original draft (equal). **Don Eslin:** Conceptualization (equal); investigation (equal); methodology (equal); writing – original draft (equal). **Derek Hanson:** Conceptualization (equal); investigation (equal); methodology (equal); writing – original draft (equal). **Michael S. Isakoff:** Conceptualization (equal); investigation (equal); methodology (equal); writing – original draft (equal). **Randal K. Wada:** Conceptualization (equal); investigation (equal); methodology (equal); writing – original draft (equal). **Virginia L. Harrod:** Conceptualization (equal); investigation (equal); methodology (equal); writing – original draft (equal). **Jawhar Rawwas:** Conceptualization (equal); investigation (equal); methodology (equal); writing – original draft (equal). **Gina Hanna:** Investigation (equal); methodology (equal); writing – original draft (equal). **William P. D. Hendricks:** Conceptualization (equal); formal analysis (equal); investigation (equal); methodology (equal); software (equal); writing – original draft (equal). **Sara A. Byron:** Formal analysis (equal); investigation (equal); writing – original draft (equal). **Matija Snuderl:** Formal analysis (equal); investigation (equal); methodology (equal); writing – original draft (equal). **Jonathan Serrano:** Formal analysis (equal); investigation (equal); methodology (equal); writing – original draft (equal). **Jeffrey M. Trent:** Formal analysis (equal); investigation (equal); methodology (equal); writing – original draft (equal). **Giselle L. Saulnier Sholler:** Conceptualization (equal); data curation (equal); formal analysis (equal); funding acquisition (equal); investigation (equal); methodology (equal); project administration (equal); supervision (equal); writing – original draft (equal); writing – review and editing (equal).

## ETHICS STATEMENT

This clinical trial was approved by the Western Institutional Review Board (WIRB) as well as by all local institutional review boards (IRB) at participating Beat Childhood Cancer (BCC) sites. Consent for study participation was obtained from all subjects according to federal and institutional guidelines. ClinicalTrials.gov Identifiers: NCT02559778.

## Supporting information


**Appendix S1**: Supplementary Information

## Data Availability

This study has been deposited in the database of Genotypes and Phenotypes (dbGaP) under accession number phs002303.v1.p1.
